# Subchronic Sleep Fragmentation Induces Endocrine Alterations and Depression-like Behavior in Male Rats

**DOI:** 10.3390/neurosci7050113

**Published:** 2026-10-21

**Authors:** Zeljko Grubač, Neriman Ezgin, Nikola Šutulović, Djurdja Jerotić, Milena Vesković, Dušan Mladenović, Emilija Đurić, Ana Stojković, Marija Matić, Aleksandra Rašić-Marković, Olivera Stanojlović, Dragan Hrnčić

**Affiliations:** 1 Institute of Medical Physiology “Richard Burian”, Belgrade University Faculty of Medicine, Visegradska 26/II, 11000 Belgrade, Serbia; 2 Department for Esophagogastric Disease, Clinic for Digestive Surgery, University Clinical Centre of Serbia, 11000 Belgrade, Serbia; 3 Department of Biotechnology, Institute of Natural and Applied Sciences, Cukurova University, 01330 Adana, Türkiye; 4 Institute of Medical and Clinical Biochemistry “Ljubisav Rakić”, Belgrade University Faculty of Medicine, 11000 Belgrade, Serbia; 5 Institute of Pathophysiology “Ljubodrag Buba Mihailovic”, Belgrade University Faculty of Medicine, 11000 Belgrade, Serbia

**Keywords:** k sleep fragmentation, hypothalamic–pituitary–adrenal axis, depression, endocrine dysregulation, rats

## Abstract

Background: Sleep fragmentation, a hallmark of obstructive sleep apnea (OSA), has been linked to mood disorders through disrupted neuroendocrine regulation. However, the time-dependent development of depression-like behaviors and hormonal changes following recurrent sleep fragmentation remains unclear. This study examined the effects of repeated experimental sleep fragmentation on depression-like behavior and endocrine status in male Wistar rats. Methods: Twenty-four male Wistar rats were randomly assigned to three experimental groups (*n* = 8 per group): treadmill control (TC; stationary treadmill), activity control (AC; 10-min running/30-min resting cycles), and sleep fragmentation (SF; 30-s running/90-s resting cycles). Treatments were applied daily for 14 consecutive days during the first 6 h of the light phase. Depression-like behavior was assessed via the forced swimming test on days 1, 7, and 14. Body weight was monitored throughout, and serum corticosterone and testosterone were measured at the endpoint. Sleep architecture was inferred from the treadmill protocol rather than directly recorded. Results: SF rats showed progressive increases in immobility and decreases in swimming and climbing over 14 days, reflecting the gradual emergence of depression-like behavior. SF animals also exhibited significantly reduced body-weight gain, elevated corticosterone (*p* < 0.01), and reduced testosterone (*p* < 0.001) compared with controls. Conclusions: Subchronic sleep fragmentation induced a progressive depression-like phenotype accompanied by neuroendocrine alterations, suggesting that recurrent sleep interruptions relevant to OSA may contribute to depression-associated endocrine dysregulation. Because sleep architecture was not directly recorded and hormones were measured only at the endpoint, these findings should be interpreted as associative rather than mechanistic.

## 1. Introduction

Obstructive sleep apnea (OSA) is a prevalent disorder characterized by recurrent upper airway obstruction leading to chronic sleep fragmentation and intermittent hypoxia [1,2]. Frequent consequences of OSA are cardiovascular, metabolic and endocrine dysfunctions, as well as mental health impairments. Namely, according to some estimations, depression occurs in approximately one-quarter to one-third of OSA patients [3]. Moreover, very recent systematic reviews and meta-analyses further indicated that OSA is an independent risk factor for depression, spotting out even a dose–response relationship between severity of OSA (apnea–hypopnea index) and depressive symptoms [4].

However, mechanisms of development and progression of depressive symptoms frequently observed in OSA patients remain poorly understood [5]. Indeed, it is quite difficult to totally distinguish between endogenous depression, mood changes and daytime sleepiness caused by OSA since there is a profound overlap of symptoms such as fatigue, poor concentration, loss of interest, and decreased libido between the two conditions. Therefore, clinical evidence indicates that depression associated with OSA often cannot be fully explained by classical psychosocial risk factors [1,6] indicating that the relationship between OSA and depression is shaped not only by psychosocial factors but also by distinct endogenous biological mechanisms [4,7].

Although there is still no strong evidence on the etiologic origin of depression in OSA patients, we could hypothesize that chronic sleep fragmentation, independent of alterations in total sleep duration and intermittent hypoxia may be one of the central pathological features implicated in the development of depressive symptoms in OSA. Namely, recurrent micro-arousals continuously activate central stress-response systems, perturbing homeostasis [8]. This persistent activation could lead to hyperactivity of the hypothalamic–pituitary–adrenal (HPA) axis and sustained elevations in glucocorticoid levels, which are recognized contributors to depression onset and development [9]. Furthermore, neuroendocrine dysregulation in OSA may extend beyond stress hormones, significantly affecting gonadal hormone balance. On the other hand, testosterone in men plays a critical role in energy homeostasis, mood and emotions, motivation, as well as stress resilience [6]. Importantly, sleep fragmentation is not unique to OSA; it is also a hallmark feature of several other sleep disorders. In particular, sleep fragmentation without hypoxia is a major manifestation of insomnia, which is as prevalent as OSA and is independently associated with increased risk of depression [10]. Additionally, sleep fragmentation is a recognized feature of periodic limb movement disorder, restless legs syndrome, narcolepsy, and various parasomnias. Therefore, the mechanistic insights derived from this study may have broader translational relevance beyond OSA.

OSA-specific chronic sleep fragmentation effects and mechanisms of alterations in glucocorticoid responses, testosterone levels, and their combined impact on depression-like behaviors remain poorly understood, as many prior studies focus on acute sleep disturbance or do not adequately isolate sleep fragmentation from confounding factors in OSA like intermittent hypoxia [11,12].

Having in mind these gaps in our understanding between OSA and depression, this study aimed to investigate how subchronic sleep fragmentation (SF), a core feature of OSA, contributes to the onset and development of depression-like behaviors and emergence of neuroendocrine imbalances. The 14-day protocol used in the present study was defined as a subchronic sleep-fragmentation protocol, and this terminology is used consistently throughout the manuscript. Herein, we specifically examined the time-dependent and cumulative effects of SF on both behavioral and hormonal parameters. Using an experimental sleep-fragmentation paradigm, behavioral changes were assessed longitudinally from acute to subchronic phases, together with endpoint measurements of serum corticosterone and testosterone.

## 2. Materials and Methods

### 2.1. Animals

Adult male Wistar albino rats (approximately 8–10 weeks of age at the start of the experiment; 180–210 g body weight) were obtained from the Military Medical Academy breeding facility (Belgrade, Serbia). Animals were group-housed according to their experimental allocation, with four rats per cage (two cages per experimental group; six cages in total). Following each experimental procedure, animals were returned to their respective group cages. Environmental conditions were controlled at 22–24 °C, 50 ± 5% relative humidity, under a 12:12 h light/dark cycle (lights on from 08:00 to 20:00). Throughout the experimental period, animals were regularly observed for general health and welfare, including changes in physical condition, behavior, and body weight. Experimental procedures were conducted with efforts to minimize unnecessary stress and suffering.

All experimental procedures complied with the European Parliament and Council Directive 2010/63/EU and were approved by the Ethical Committee of the School of Medicine, University of Belgrade under Permission No. 323-07-05290/2017-05.

### 2.2. Experimental Design and Sleep-Fragmentation Protocol

This study was designed as a randomized controlled preclinical experimental animal study with three parallel groups and repeated behavioral assessments. A treadmill-based protocol designed to produce repeated sleep interruption was applied according to the method of McKenna et al. [13] during the first 6 h of the light phase. This time window was selected to maintain consistency with the established treadmill-based sleep-fragmentation protocol described by McKenna et al. [13]. Animals were randomly assigned to the three experimental groups (*n* = 8 per group) using a computer-generated randomization table. The procedure was conducted for 14 consecutive days, with behavioral assessments using the Forced Swimming Test (FST) performed on days 1, 7, and 14. The primary outcomes of the study were behavioral parameters assessed by the forced swimming test and serum testosterone and corticosterone levels. Body weight was measured daily at 8:00 a.m. for each animal.

The treadmill apparatus (NeuroSciLaBG-Treadmill, Elunit, Serbia) consisted of a conveyor belt with programmable motion. Treadmill activity was defined as ON mode (belt moving forward at 0.02 m/s) and OFF mode (belt stationary at 0.00 m/s). Animals were acclimated to the treadmill for 2 days prior to the experiment, with 1-h sessions alternating between 5 min ON and 5 min OFF cycles.

Sleep fragmentation intervals were designed to mimic the sleep pattern of apneic patients. Each hour was divided into 30 s intervals of 2 min, with alternating treadmill activity. Accordingly, the treadmill was programmed to operate in cycles of 30 s ON and 90 s OFF for 6 h during the light phase each day for 14 consecutive days (Sleep Fragmentation group, SF; *n* = 8).

The activity control group (AC; *n* = 8) was exposed to treadmill motion matching the total movement of the SF group but without sleep fragmentation, using cycles of 10 min ON and 30 min OFF. Treadmill control rats (TC; *n* = 8) were placed in the apparatus without belt movement (0 m/s), under conditions equivalent to their home cages.

**Figure 1:**
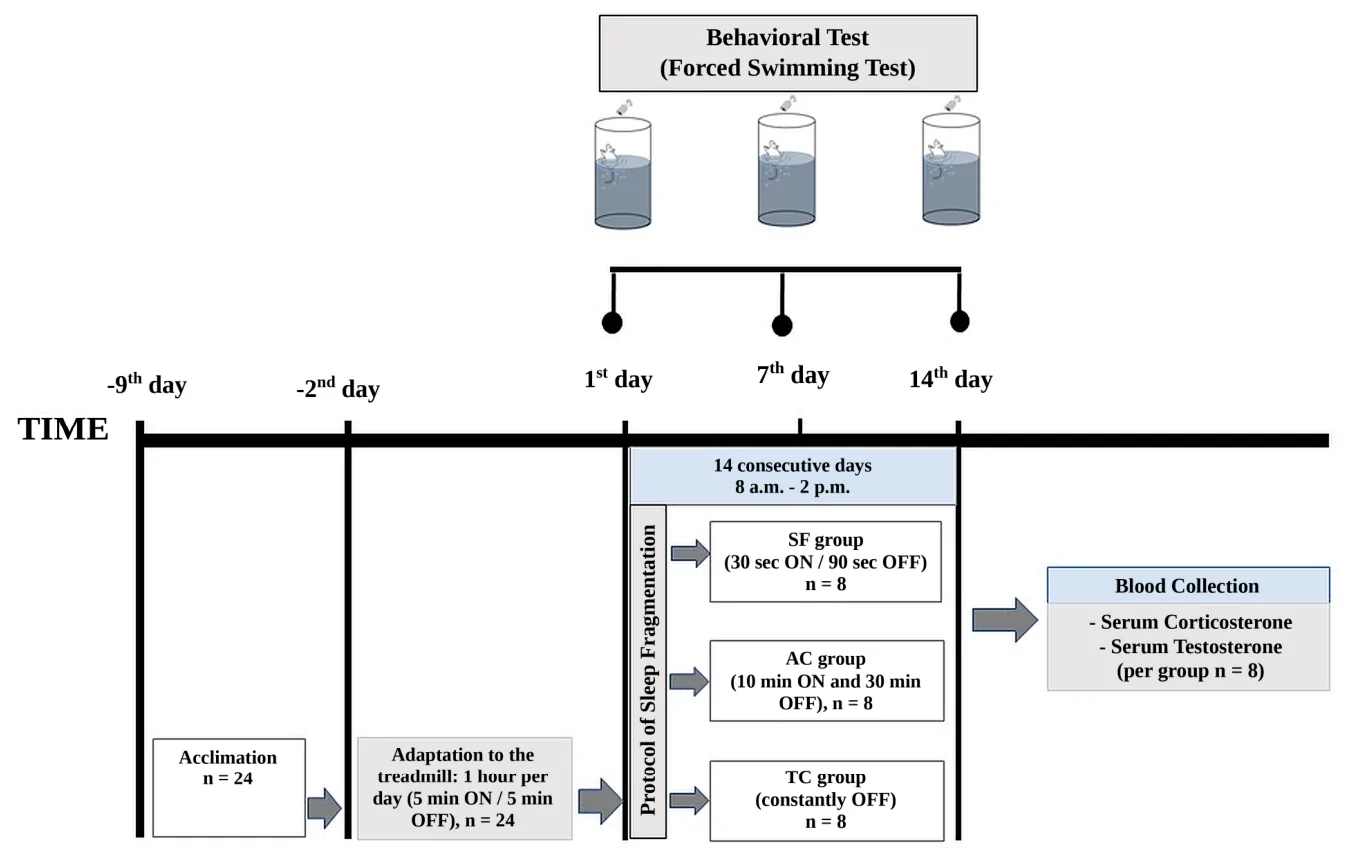
Study Design. Experimental sleep fragmentation paradigm was induced using a motorized treadmill during the first 6 h of the light phase for 14 consecutive days. Animals were acclimated to the treadmill for 2 days prior to the experiment with 1 h sessions, alternating 5 min ON and 5 min OFF. Rats were assigned to three groups: sleep fragmentation (SF, *n* = 8), activity control (AC, *n* = 8), and treadmill control (TC, *n* = 8). In the SF group, treadmill activity alternated between 30 s ON and 90 s OFF cycles to induce sleep fragmentation. The AC group received matched total treadmill activity using 10 min ON/30 min OFF cycles without disrupting sleep. The TC group remained on a stationary treadmill (0 m/s) under conditions equivalent to standard housing. Behavioral testing using the forced swimming test (FST) was performed on days 1, 7, and 14. Body weight was measured daily at 08:00. At the end of the experiment (day 14), blood samples were collected for serum testosterone and corticosterone analysis.

At the end of the 14-day experiment, blood samples were collected for hormone analysis, including testosterone and corticosterone. No EEG or EMG recordings were performed in the present study. Accordingly, sleep fragmentation was inferred from the treadmill-based stimulation protocol and previous experience rather than electrophysiologically confirmed. Throughout the manuscript, the term “sleep fragmentation” refers to the experimental sleep-fragmentation paradigm and should not be interpreted as direct evidence of quantified changes in sleep architecture. Therefore, in the present study, sleep fragmentation refers to the experimental treadmill-based paradigm and not to electrophysiologically quantified alterations in sleep architecture. The experimental design and sleep fragmentation protocol are presented in detail in Figure 1. No animals or data points were excluded from the analysis.

### 2.3. Forced Swimming Test

The forced swimming test (FST) was conducted following the established protocol of Porsolt [14]. Rats were individually placed in cylindrical plastic containers (50 cm height × 25 cm inner diameter) filled with 35 cm of water maintained at 22 ± 1 °C, and their behavior was continuously video-recorded for 5 min for subsequent analysis. Behavioral responses were classified into three categories: Struggling, defined as vigorous climbing or thrashing with all four limbs actively engaging the water surface; Swimming, defined as coordinated horizontal movements or active propulsion using the limbs; and Floating, characterized by minimal movement while maintaining the nose above water. The duration of each behavior was quantified in seconds for each animal, and relative proportions were calculated. To minimize observer bias, all video scoring was performed by a single investigator blinded to the experimental groups. Rats were acclimated to the FST apparatus for 15 min on two consecutive days prior to the first test session. Initial and baseline measurements have been done on day 0. Afterwards, testing was performed immediately following the treadmill session on days 1, 7, and 14.

### 2.4. Biochemical Hormone Level Determination

On day 14, following completion of the treadmill session and the subsequent FST, animals were sacrificed by decapitation 2 h later and blood samples were collected for hormone analysis. Blood collection was performed at the same circadian time across all experimental groups, i.e., at 16 h. Hormone measurements were restricted to this terminal time point because the endocrine assessment was designed as an endpoint analysis of the 14-day experimental protocol; consequently, longitudinal changes in hormone concentrations were not assessed. Blood samples were collected and centrifuged at 1575× *g* for 10 min. Serum was separated and stored at −80 °C until hormone analysis. Testosterone and corticosterone levels were measured using commercially available enzyme-linked immunosorbent assay (ELISA) kits (Cusabio Technology LLC, Houston, TX, USA, for testosterone: cat. no. CSB-E05100r, detection Range: 0.13 ng/mL–25.6 ng/mL, densitivity: 0.06 ng/mL and for corticosterone: CSB-E07014r, detection range: 0.2 ng/mL–40 ng/mL, sensitivity: 0.1 ng/mL) according to the manufacturer’s instructions. All samples were assayed in duplicate. These analyses are routinely performed in our laboratory.

### 2.5. Statistical Analysis

Normality of data distribution was assessed using the Shapiro–Wilk test and behavioral and biochemical data were found to be normally distributed. Accordingly, all results are presented as mean ± standard deviation (SD). Sample size was determined based on previous studies using similar experimental designs, as no formal a priori power calculation was performed. Between-group comparisons at individual time points were performed using one-way analysis of variance (ANOVA) followed by the Tukey post hoc test, where appropriate. Repeated-measures ANOVAs were used to test statistical significance of within-group differences at different time points. Statistical significance was defined as *p* < 0.05, *p* < 0.01, or *p* < 0.001.

## 3. Results

### 3.1. Sleep Fragmentation Reduced Body-Weight Gain

**Figure 2:**
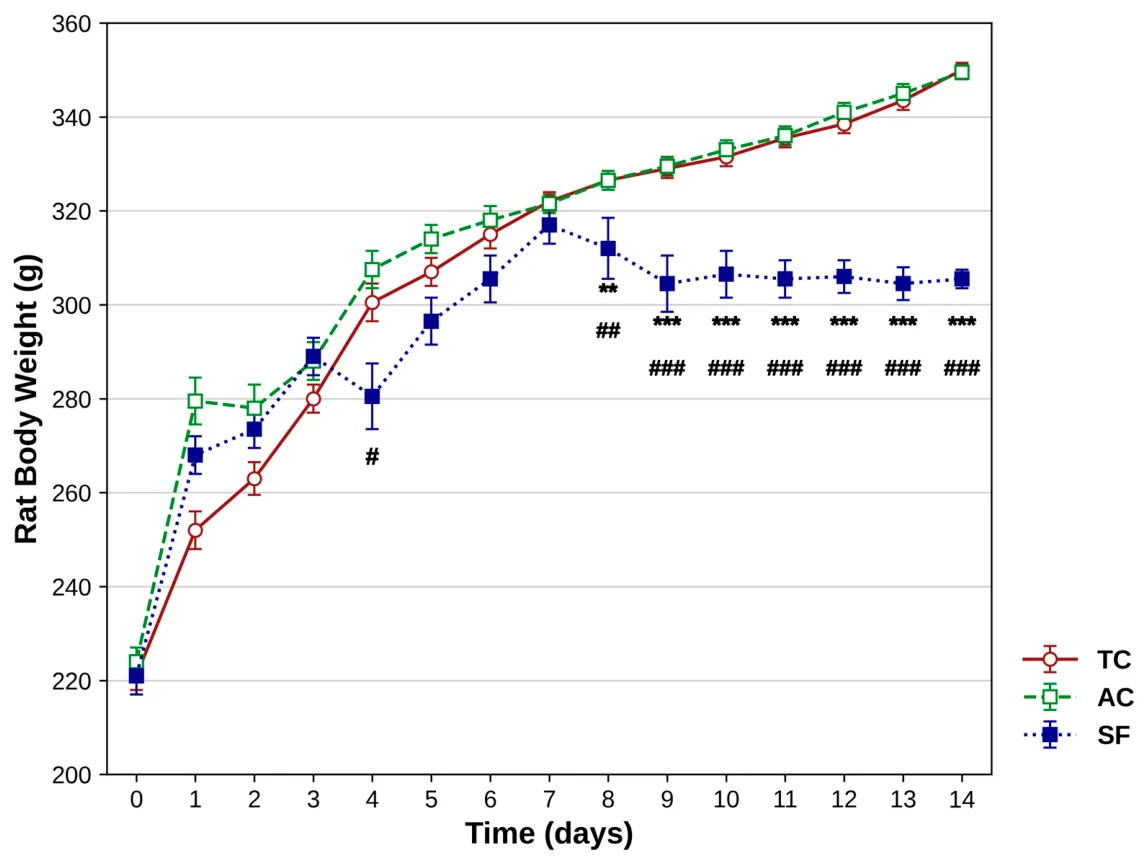
Rat body weight during 14 days of experiment. TC: Treadmill control group—consisted of rats staying in the treadmill apparatus at moving speed of 0 m/s and conditions equivalent to those in cages. AC: Stress “activity” control group—treadmill was programmed to work in cycles of 10 min ON and 30 min OFF. SF: Sleep fragmentation group—the treadmill alternately worked for 30 s ON and 90 s OFF. Between-group differences were estimated by one-way ANOVA with Tukey post hoc test. ** *p* < 0.01, *** *p* < 0.001 vs. AC # *p* < 0.05, ## *p* < 0.01, ### *p* < 0.001 vs. TC.

Body weight increased over the experimental period in all groups; however, the SF group exhibited reduced body-weight gain compared with the TC and AC groups, with significant between-group differences observed from day 8 onward (*p* < 0.001) (Figure 2). This attenuated body-weight gain should be considered as a potential confounding factor when interpreting the behavioral results, as differences in body condition may independently affect performance in the forced swimming test.

### 3.2. Forced Swimming Test (FST) Revealed Increased Depression-like Behavior in SF Group

**Figure 3:**
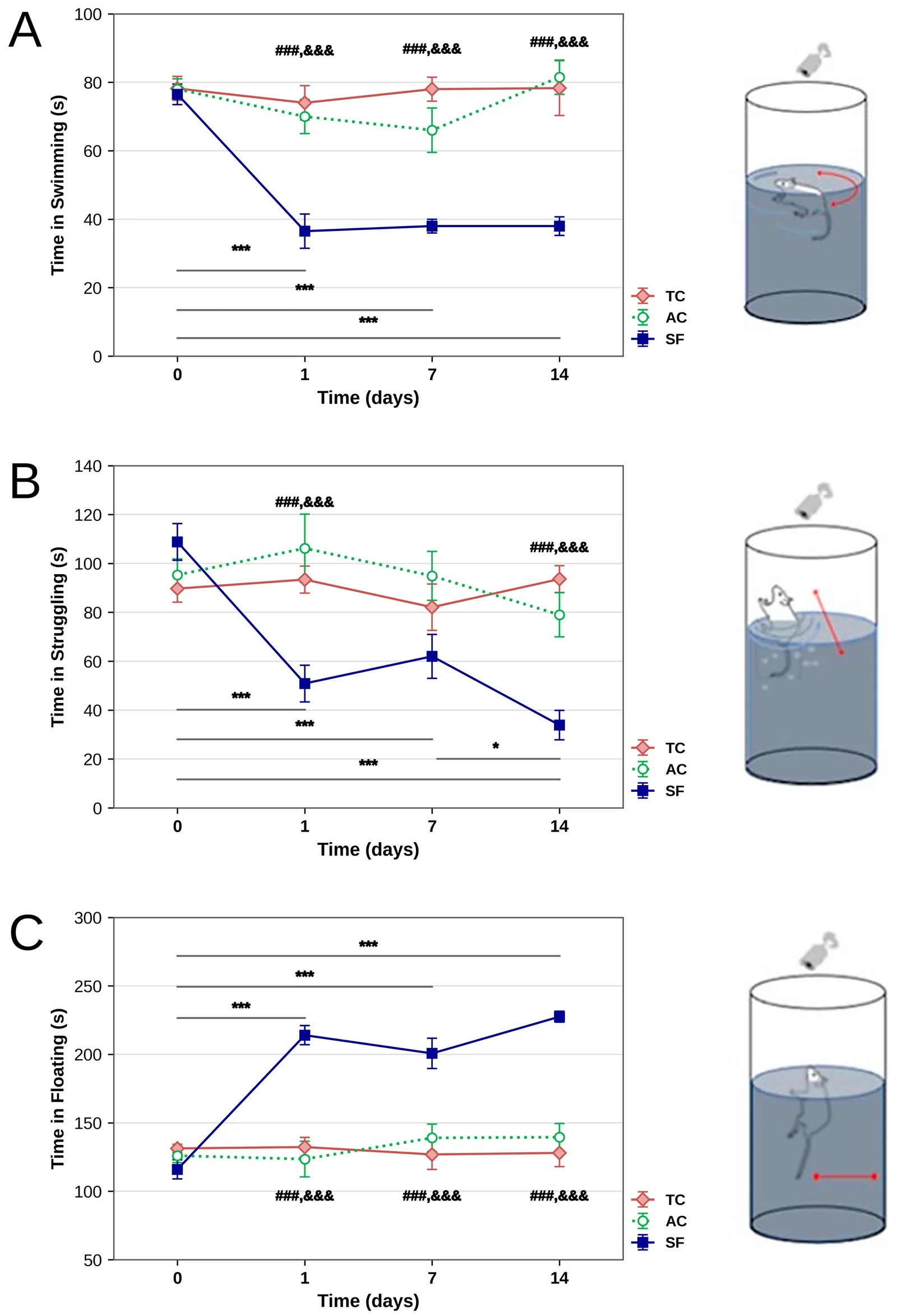
The effects of the subchronic experimental sleep fragmentation paradigm on depression-like behavior. Distribution and duration of swimming (**A**), struggling (**B**) and floating (**C**) in animals from the experimental (SF) and control groups (TC and AC) in the forced swim test (FST). The graphs show the duration of swimming, struggling and floating in animals from all three groups at baseline (day 0) and following 1, 7 and 14 days of the sleep fragmentation protocol. The significance of differences between groups was determined using one-way ANOVA with Tukey post hoc test (### *p* < 0.001 vs. TC; &&& *p* < 0.001 vs. AC), while within-group differences were estimated by repeated measures ANOVA (* *p* < 0.05, *** *p* < 0.001). Data are presented as mean ± SD.

Floating time was significantly increased in the SF group compared with TC and AC groups across all time points (SF: day 0, 114.50 ± 18.72 s; day 1, 212.63 ± 19.96 s; day 7, 199.75 ± 29.82 s; day 14, 228.13 ± 11.93 s; TC: day 0, 131.88 ± 9.42 s; day 1, 132.50 ± 20.06 s; day 7, 127.38 ± 32.43 s; day 14, 128.00 ± 27.41 s; AC: day 0, 126.75 ± 17.72 s; day 1, 124.00 ± 39.27 s; day 7, 139.00 ± 28.31 s; day 14, 139.38 ± 29.84 s; *p* < 0.001). Struggling time in the SF group was significantly reduced on days 1 and 14 compared with TC and AC groups (SF: day 0, 109 ± 20.74 s; day 1, 51.00 ± 20.77 s; day 7, 62.13 ± 25.94 s; day 14, 33.88 ± 16.13 s; *p* < 0.001), with no difference observed on day 7. A progressive decline was evident in the SF group, reaching significance on day 14 compared with baseline (day 0, *p* < 0.001). Similarly, swimming time in the SF group was significantly decreased at all points relative to TC and AC (SF: day 0, 76.50 ± 8.62 s; day 1, 36.38 ± 13.03 s; day 7, 38.13 ± 5.19 s; day 14, 38.00 ± 7.05 s; *p* < 0.001) (Figure 3). Notably, behavioral differences between the SF group and controls were already evident on day 1, suggesting that both acute stress responses and cumulative effects of repeated sleep fragmentation may contribute to the observed phenotype.

### 3.3. Sleep Fragmentation Increased Serum Corticosterone and Decreased Serum Testosterone Concentration

**Figure 4:**
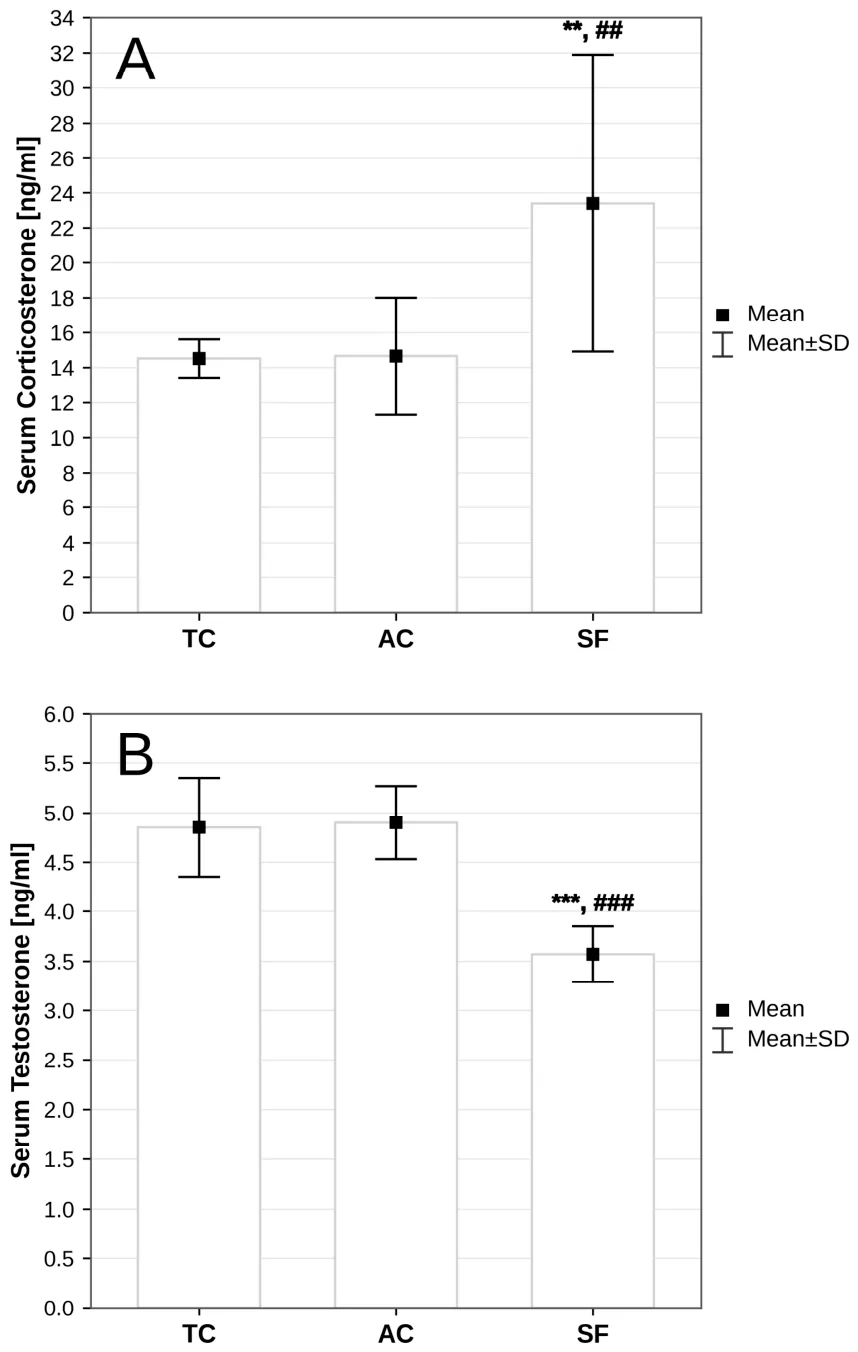
The effects of sleep fragmentation (SF) on serum hormone concentrations. Serum corticosterone concentration (**A**) and serum testosterone concentration (**B**). The within-group differences were estimated by one-way ANOVA with Tukey post hoc test. ## *p* < 0.01 and ### *p* < 0.001 vs. TC group; ** *p* < 0.01, *** *p* < 0.001 vs. AC, *p* < 0.05, *p* < 0.001 vs. TC group. Data is presented as mean ± SD.

Serum corticosterone levels were significantly elevated in the SF group (23.43 ± 8.51 ng/mL) compared with both TC (14.55 ± 1.09 ng/mL) and AC groups (14.68 ± 3.36 ng/mL; *p* < 0.01) (Figure 4A). In contrast, SF induced a marked reduction in serum testosterone levels (3.57 ± 0.28 ng/mL) relative to TC (4.86 ± 0.50 ng/mL) and AC groups (4.90 ± 0.37 ng/mL; *p* < 0.001) (Figure 4B).

## 4. Discussion

Results of our study showed that rats exposed to SF for 14 days exhibited behavioral changes consistent with a depression-like phenotype, displaying increased immobility/floating and decreased swimming and struggling times in the FST. These behavioral disturbances were accompanied by elevated corticosterone and reduced testosterone levels, consistent with HPA axis hyperactivity and endocrine dysregulation. A major limitation of the present study is the absence of EEG/EMG recordings. Consequently, the protocol-induced changes in sleep–wake states, arousal frequency, total sleep time, and sleep architecture could not be objectively quantified. Thus, although the treadmill paradigm was designed to produce repeated sleep interruption based on a previously established protocol, the actual degree of sleep fragmentation remains unverified electrophysiologically. The behavioral and endocrine findings should therefore be interpreted as responses associated with the experimental sleep-fragmentation paradigm rather than with objectively quantified sleep fragmentation.

FST measurements obtained at three time points indicated an overall pattern of increased immobility and decreased active coping behaviors in the SF group relative to controls. Notably, behavioral differences were already detectable on day 1, suggesting that both acute stress responses and cumulative effects of repeated sleep fragmentation may underlie the observed phenotype, rather than a purely progressive, cumulative process. Yin et al. (2024) showed that sleep deprivation impairs cognitive function and leads to depression-like behaviors by activating the TLR4/MyD88/NF-κB signaling pathway, while melatonin treatment alleviates these neuroinflammatory responses [15]. Zhang et al. (2025) systematically reviewed sleep deprivation in rats and mice, showing that sleep disturbances impair memory function and increase immobility duration in the Forced Swimming Test [16]. These findings are broadly consistent with the behavioral pattern observed in our sleep fragmentation model. Moreover, Yan et al. (2023) also reported depression-like phenotypes in a distinct chronic SF model [17]. Nevertheless, the forced swim test primarily reflects stress-coping behavior rather than a comprehensive depressive phenotype, and this interpretive limitation should be considered when generalizing our findings. In the FST, increased floating (immobility), together with reduced active behaviors such as swimming and struggling, was interpreted as a depression-like behavioral pattern within the context of this test. However, behavior was not systematically assessed after the animals were returned to their home cages; therefore, the present study cannot determine whether these behavioral alterations persisted outside the FST context. Future studies incorporating additional behavioral assays such as the sucrose preference test, open field test, and elevated plus maze would provide a more comprehensive characterization of the affective phenotype. Results of hormonal analysis in our study showed that rats undergoing repeated sleep fragmentation over a period of 14 days exhibited significant changes in hormonal levels, with elevated corticosterone and reduced testosterone levels in comparison with corresponding controls. Serum corticosterone concentration in the SF group was significantly higher compared to the TC and AC groups, consistent with increased HPA axis activity. Although the magnitude of corticosterone elevation observed here (~23 ng/mL in SF vs. ~14–15 ng/mL in controls) is more modest than that reported in some acute stress paradigms, comparable elevations have been described in subacute rodent stress models without additional stressors such as hypoxia [18]. Previous studies have shown that both acute and chronic corticosterone exposure increases alertness and activates the HPA axis [19]. Similarly, acute SF has been reported to rapidly increase corticosterone and pro-inflammatory cytokines [20]. Repeated corticosterone administration has also been shown to elicit depression-like behaviors in rodents [21], and sleep deprivation has been reported to alter neurotransmitter receptor sensitivity and neuroendocrine stress responses in association with behavioral deficits [11]. Sleep fragmentation has additionally been linked to increased pro-inflammatory cytokines and decreased brain-derived neurotrophic factor (BDNF) expression in other experimental models [21,22,23]. While these mechanisms offer plausible pathways linking HPA axis activation to depression-like phenotypes, they were not directly assessed in the present study, and their involvement here remains speculative. Furthermore, because corticosterone was measured only at a single terminal time point, the temporal relationship between HPA axis activation and the behavioral changes observed across the 14-day protocol cannot be established from the present data. In addition, because terminal blood sampling on day 14 was performed 6 h after the FST, the acute stress response elicited by forced swimming may have contributed to the measured hormone concentrations, particularly corticosterone. All groups underwent the same protocol and were acclimatized to the FST test in two points. However, this potential confounding effect should be considered when interpreting the hormonal findings.

Simultaneously, a significant decrease in testosterone levels was observed in the SF group following 14 days of sleep fragmentation. Reductions in testosterone have similarly been observed following short-term sleep fragmentation, in association with anxiety-like behaviors and androgen deficiency [24]. Sleep deprivation has also been reported to alter testicular structure [25] and impair male reproductive function [26], which may partly account for the testosterone decrease observed in our study. Gonadal hormones have been shown to modulate sleep–wake behavior in a sex-specific manner [27], consistent with a possible contribution of testosterone deficiency to the behavioral and mood outcomes observed here. Corticosterone and testosterone are known to be functionally interdependent, as corticosterone can suppress gonadal steroidogenesis [28], and stress-induced gonadotropin inhibitory hormone release may further restrict testosterone production [29]. This HPA-HPG interaction offers a plausible, literature-based explanation for the concomitant endocrine changes observed in our SF rats, although this interaction was not directly tested in the present study. Reduced testosterone levels may contribute to depression-like behaviors through effects on motivation, reward circuits, and neurotrophic signaling in the limbic system [30]; however, the present study did not directly assess reproductive axis signaling or test a causal role for testosterone in the observed behavioral phenotype, and this interpretation therefore remains associative. Male rats were selected because testosterone was a predefined endocrine outcome of the study and the experimental design specifically focused on the relationship between sleep fragmentation, testosterone alterations, and depression-like behavior. Nevertheless, the exclusive use of male animals represents an important methodological limitation. Sex differences in HPA axis reactivity, sleep architecture, and depression susceptibility are well documented, and the generalizability of these findings to females cannot be assumed. Future studies should include female subjects to characterize sex-specific responses to sleep fragmentation. Collectively, these findings suggest that SF is associated with concurrent neuroendocrine, hormonal, and behavioral changes, and that this model may serve as a useful, though preliminary, tool for investigating mood disturbances associated with disrupted sleep.

As noted above, the reduced body-weight gain observed in the SF group may itself have acted as a confounding factor influencing FST performance, independent of any direct effect of sleep fragmentation on mood-related behavior; this possibility should be considered when interpreting the behavioral results. The mechanisms underlying the body-weight changes observed in the SF group cannot be determined from the present study because food intake, energy expenditure, and metabolic parameters were not directly measured. Therefore, altered food intake, increased energy expenditure, metabolic changes, or other physiological consequences of the experimental protocol remain possible explanations rather than established mechanisms. Furthermore, because total sleep time and sleep architecture were not directly measured, the present data cannot determine whether similar body-weight changes would occur if total sleep duration were preserved while only sleep continuity was disrupted. The literature reports that short-term sleep apnea alters metabolic hormones such as leptin and ghrelin by affecting energy homeostasis and HPA axis activity [31]. Furthermore, sleep disruption has been observed to increase energy expenditure and decrease metabolic efficiency, consistent with weight loss trends in the SF group [32]. Hippocampal metabolomic studies show that SF affects amino acids, energy substrates, and neurotransmitter-related metabolites, disrupting central energy regulation [33]. These central metabolic changes parallel peripheral weight loss and metabolic dysregulation. Disruptions in hippocampal energy metabolism have also been reported to have a direct impact on neurogenesis and energy balance [34]. Long-term SF exposure has been found to cause persistent imbalances in metabolic and cardiovascular systems [35], supporting the cumulative physiological stress observed after the 14-day protocol.

The neuroendocrine and behavioral changes observed in the SF model parallel those in OSA in humans. In male patients with moderate to severe OSA, the HPA and hypothalamus–pituitary–gonadal (HPG) axes are disrupted, serum testosterone levels decrease, and testosterone/cortisol ratios change [6,9]. Low testosterone levels have been associated with increased depressive symptoms in OSA patients, supporting the link between androgen deficiency and mood disorders [6]. While the Forced Swim Test is commonly used to assess depression-like behaviors, it primarily reflects coping with stress and behavioral hopelessness. Therefore, incorporating additional behavioral paradigms will enhance the interpretation of SF’s effects on mood.

Dysfunction of the HPA axis has been commonly reported in patients with OSA, and observations of impaired diurnal cortisol secretion, with partial normalization following continuous positive airway pressure (CPAP) therapy, further support the pathophysiological relevance of stress responses in this context [36].

Additionally, clinical studies have shown that increasing OSA severity is associated with greater intensity of anxiety and depressive symptoms [37], consistent with a reciprocal relationship between disrupted sleep and mood disorders. Immune and inflammatory mechanisms have also been increasingly implicated in the pathophysiology of OSA [38]. These clinical observations lend some translational relevance to our subchronic SF rat model as a paradigm for exploring the relationship between sleep continuity and neuroendocrine and emotional outcomes. Importantly, the present experimental paradigm was designed to examine repeated sleep fragmentation in isolation and should not be regarded as a complete model of OSA. Clinical OSA is a multifactorial disorder characterized not only by recurrent sleep fragmentation but also by upper-airway obstruction and intermittent hypoxia, neither of which was reproduced in the present study. Therefore, the current findings specifically address behavioral and endocrine alterations associated with the experimental sleep-fragmentation paradigm and cannot be extrapolated to the full pathophysiology of OSA. Nevertheless, because recurrent sleep fragmentation is also observed in OSA and in several other sleep disorders, the present findings may provide information relevant to the consequences of disrupted sleep continuity, while their direct applicability to OSA remains limited.

Many prior studies, including our previous studies focus on acute sleep disturbance. Herein, in a separate cohort of animals, we addressed the effects of subchronic sleep fragmentation and mechanisms of alterations in glucocorticoid responses and testosterone levels on depression-like behaviors.

Our findings suggest that recurrent sleep fragmentation is associated with endocrine alterations and depression-like behaviors independently of hypoxic stress, pointing to sleep continuity as a potentially important determinant of mood regulation. However, given the methodological limitations discussed above—including the absence of direct sleep architecture verification, single-time-point endocrine sampling, and reliance on a single behavioral assay—these findings should be interpreted as associative rather than establishing a confirmed mechanistic or causal pathway. An additional consideration is that repeated daytime treadmill stimulation for 14 days may have altered circadian phase relationships; because corticosterone, testosterone, and behavioral stress responses are under strong circadian regulation, some of the observed changes may reflect circadian misalignment rather than, or in addition to, sleep fragmentation per se. This possibility cannot be fully excluded with the current design and represents a limitation that future studies should address through circadian phase assessment and clarification of sampling timing relative to the light/dark cycle.

## 5. Conclusions

Repeated experimental sleep fragmentation was associated with endocrine and behavioral alterations in male rats, including changes in body weight, elevated corticosterone, reduced testosterone, and behavioral changes consistent with a depression-like phenotype. Although recurrent sleep fragmentation is a clinically relevant feature of OSA and other sleep disorders, the present experimental paradigm does not reproduce the full pathophysiology of OSA, particularly intermittent hypoxia and upper-airway obstruction. Therefore, these findings should be interpreted specifically in the context of experimentally induced repeated sleep interruption rather than as effects of OSA itself. HPA axis hyperactivation and androgen suppression emerge as plausible contributors to the affective changes observed, offering a preliminary translational model for exploring the relationship between fragmented sleep and neuroendocrine-behavioral outcomes. However, given the methodological limitations of this study—including the absence of polysomnographic confirmation of sleep fragmentation, single time-point endocrine sampling, use of only male rats, reliance on a single behavioral assay, and the potential confounding influence of body weight loss and circadian phase alterations—these findings should be regarded as preliminary and hypothesis-generating rather than establishing a confirmed causal or mechanistic link. Future studies incorporating direct sleep architecture monitoring, longitudinal hormone profiling, complementary behavioral paradigms, and both sexes will be needed to more rigorously characterize the mechanisms linking sleep fragmentation to affective and endocrine dysregulation.

## Data Availability

The original contributions presented in this study are included in the article. Further inquiries can be directed to the corresponding author.
